# Relationships between Difficulty with Healthcare Services and Psychosocial Adjustment in Hispanic Older Adults

**DOI:** 10.1093/geroni/igaf122.2711

**Published:** 2025-12-31

**Authors:** Atami De Main, Daphne Buitron, Irina Mindlis

**Affiliations:** Weill Cornell Medicine, New York, New York, United States; Weill Cornell Medicine, New York, New York, United States; Rutgers University, New York, New York, United States

## Abstract

Living with multiple chronic conditions (MCC) is now the norm for older adults in the U.S. (77%), and requires adjustments across multiple life domains, including maintaining quality of life (QOL) and protecting mental health. Among older adults with MCC, depressive symptoms and poor QOL increase risk for morbidity and mortality. Hispanic/Latino older adults are more likely to sustain early onset of MCC, report poor self-rated health, and experience significant difficulties accessing health care. This study aimed to investigate the relationships between difficulties accessing healthcare services and psychological adjustment, specifically depressive symptoms and QOL, among Hispanic/Latino older adults. Data were collected from 118 participants (ages 62-92; Mean=73.6, SD = 6.33) at senior centers in New York City between July 2024 and February 2025. The sample was diverse, with 42.9% identifying as Puerto Rican, 21.4% as Dominican, and 20.4% as South American. Common chronic conditions reported included hyperlipidemia (70.7%), arthritis (65.2%), hypertension (64.1%), diabetes (51.1%), and asthma (30.4%). The results of the regression analysis indicated that poorer health status and difficulties with healthcare services were significantly associated with worsened QOL controlling for demographic factors (R2adj=0.35, F(9, 83) = 5.25, p<.001). In contrast, high depressive scores did not significantly correlate with difficulties accessing healthcare services (R2adj=0.11, F(9, 83) = 0.99, p = 0.45). These findings underscore the critical role of healthcare access in promoting the mental health and well-being of Hispanic/Latino older adults with MCC, highlighting the need for further research into the mechanisms linking MCC to poor psychosocial adjustment.

